# The Role of Bedside Medication Delivery in Reducing Hospital Readmissions: A Quality Improvement Study

**DOI:** 10.7759/cureus.109158

**Published:** 2026-05-18

**Authors:** Alexander O'Hara, Mohammed Abourahma, Moujtaba Y Kasmani, Dinkar Ahuja, Michael Wesolowski, Joseph Hegazin, Ryan Mayhew, Agnes R Libot

**Affiliations:** 1 Internal Medicine, Loyola University Medical Center, Maywood, USA; 2 Biostatistics, Loyola University Medical Center, Maywood, USA; 3 Pharmacy, Loyola University Medical Center, Maywood, USA

**Keywords:** discharge medication, hospital readmission, medication adherence strategies, medication delivery, transition of care

## Abstract

Transitions of care place patients at elevated risk for adverse events that may result in hospital readmission, including medication non-adherence after discharge. We sought to apply principles of quality improvement to improve medication adherence after hospitalization by implementing a pharmacist-led bedside medication delivery program at the time of hospital discharge. Our program was not associated with reduced 30-day hospital readmissions in the one-year period analyzed. We further stratified patients by demographic characteristics, insurance status, and zip code to better identify those at elevated risk of readmission or ineligible for enrollment in the program. After comparing our results with similar programs reported in the literature, we postulate that isolated bedside medication-delivery programs, as presently implemented, do not meaningfully reduce hospital readmissions in most cases. There is some evidence that these programs may benefit from being bundled with multi-faceted interventions in the future.

## Introduction

Medication non-adherence is a well-recognized problem after hospital discharge. Many studies have examined rates and impacts of medication non-adherence after discharge; a 2021 meta-analysis analyzing 8949 patients found that non-adherence ranged from 7.0% to 83.5% (pooled prevalence = 42.6%) with significant heterogeneity stemming from patient comorbidity burden and demographic characteristics [1]. Medication non-adherence has been found to contribute to 33%-69% of medication-related hospital readmissions with associated costs of nearly one hundred billion dollars a year [2]. Physicians have been found to be poor estimators of medication adherence after discharge [3]. Extensive analyses of efforts to reduce non-adherence and improve transitions of care have found that many strategies are overly complex and ineffective at improving both adherence and treatment outcomes [4].

In light of these persistent challenges, and within the national framework of quality-driven payment models adapted by the Centers for Medicare and Medicaid Services (CMS), such as the Hospital Readmissions Reduction Program (HRRP), the practice of quality improvement has been applied by physician and pharmacist-led teams in various settings to attempt to improve medication adherence at time of discharge and decrease downstream negative impacts, including hospital readmissions. 

Quality improvement in healthcare focuses primarily on two mechanisms: systematic problem-solving to decrease variability and adherence to evidence-based practices through established guidelines and protocols using structured frameworks such as the Plan-Do-Study-Act (PDSA) cycle [5-7]. Standardization and the perspective of problems as system-wide issues aim to minimize human error and randomness and facilitate evidence-based decision-making that enhances patient outcomes [8]. Quality improvement initiatives have demonstrated substantial benefits across various healthcare domains and are now commonly applied to systems that are felt to be underperforming or necessitating optimization. Given the proven benefits of standardization and systematic problem-solving, the “meds-to-beds” (M2B) initiative was derived from quality improvement practices at our center and resulted in a program that implemented bedside medication delivery directly to patients on the day of discharge.

Similar programs have been reported in the medical literature with varying results. A 2019 meta-analysis, which examined nearly all previously reported bedside medication delivery programs, concluded that data regarding their utility, including their impact on 30-day readmission rates, optimal program design, and which patients may most benefit, remains insufficient [9]. As prior results have been heterogeneous, we hope that the findings generated by this observational study contribute to the growing body of work that describes the efficacy of such programs. Some prior studies have stratified patients by their comorbidity burden and demographic characteristics; fewer have examined the roles of other patient-specific variables such as insurance status or specifically hailing from an underserved population. Presently available data on bedside medication delivery programs remain insufficient for drawing conclusions that are generalizable to the healthcare system as a whole. This article was presented as a meeting abstract at the 2025 Society of General Internal Medicine Annual Meeting on May 16, 2025, in Hollywood, Florida.

## Materials and methods

Setting and patient population

Our hospital is a 547-bed academic medical center located in Maywood, Illinois, on the outskirts of Chicago that anchors a quaternary care healthcare system. This study was confined to the main medical center campus and was not extended to affiliated hospitals or satellite sites. On hospital discharge, patients typically elect to have their medications sent to a community pharmacy of their choice or to the hospital’s outpatient retail pharmacy. The hospital pharmacy is open from 8 AM to 6 PM Monday to Friday and is closed on weekends. It is staffed by a team of fully licensed and resident pharmacists. This intervention was approved by the Institutional Review Board (IRB#: 218638) at our institution. All patient-specific data were fully anonymized.

Patient selection 

The M2B program at our center was first implemented on a single general medicine floor in 2016 and has since grown to provide coverage to 12 medical and surgical units. The covered floors contain patients under the care of nearly every service in the hospital. All adult patients (>18 years of age) discharged to their home from any of the medical and surgical floors were included in the final analysis. Patients discharged directly from an intensive care unit, pediatric patients, and oncology patients were excluded, as their discharge medications are processed by separate pharmacy teams. Patients discharged to community nursing and long-term care facilities with on-site medication dispensing capabilities were also excluded. We examined all eligible discharges over the approximately one-year period ranging from 11/13/22 to 12/31/23 to assess the function of the program in a mature state. 

Intervention 

A prompt labeled “Ask,” within an M2B-specific dashboard in the electronic medical record (EMR), was implemented in the pharmacy-specific EMR context. This is used by all pharmacy staff. Pharmacy technicians, who received standardized training regarding the M2B program, meet with every patient on eligible units and ask whether they would like to enroll in the M2B program between 8 AM Monday and 5 PM Friday. Patients may respond “Yes” or “No,” which is then updated to their final enrollment status in the EMR. Technicians attempt to ask each patient twice before recording a final status as “No.” Other possible final status updates include the following: “ineligible” if not discharging to home; “unable to ask” if a patient did not have decision-making capacity and an appropriate representative could not be reached, or they spent such significant time off-unit that the technician did not make contact with them; and “not recorded” if their status was never updated before discharge, typically due to overnight observation or weekend admissions. 

When a patient elects to enroll in the program, the pharmacy technician updates their preferred pharmacy to “in hospital outpatient pharmacy” (which corresponds to the local outpatient pharmacy (LOP)), updates patient medication allergies, and obtains insurance information. Prior authorizations are initiated if necessary. At the time of discharge, providers electronically send prescriptions to LOP. The patient's medication summary is reviewed and processed by a pharmacist. Patients whose status was updated to “Yes” in the M2B dashboard then have their medications delivered to the bedside by a pharmacy technician. Patients are able to ask the technician any questions, which can be escalated to a pharmacist on duty. Additional medication counseling is provided by the patient's primary nurse before discharge. Pharmacists then update a separate prompt in the dashboard to confirm medication delivery or note any barriers to delivering medications. Medical teams are encouraged to anticipate weekend discharges and to work with pharmacy staff to ensure that enrolled patients receive their medications before the end of the week when necessary. 

Study outcomes 

Primary study outcomes were 30-day all-cause inpatient hospital readmissions to assess the program’s impact on commonly utilized quality metrics. Secondary outcomes focused on identifying which patients may be at elevated risk of readmission and which were more or less likely to enroll in the program based on demographic characteristics. 

Statistical analysis 

Response values of “unable to ask” were categorized as “No” for M2B enrollment. Values of “ineligible” and “not recorded” were coded as missing. Summary statistics were generated to describe the patient sample of this cross-sectional study. Frequencies and percentages are reported for categorical variables. Medians and interquartile ranges are reported for quantitative variables. Row/column percentages and stratified frequencies, medians, and interquartile ranges are additionally reported to describe bivariate relationships. Pearson’s Chi-square tests for independence were used to evaluate bivariate associations between categorical variables. Wilcoxon rank sum tests were used to evaluate bivariate associations between binary and quantitative variables. Sensitivity analyses for missing data were considered outside of the scope of this observational study and were not performed. p-values less than 0.05 were considered statistically significant. 

## Results

Between 11/13/2022 and 12/31/2023, 5068 patients were hospitalized at Loyola University Medical Center (LUMC). Of these, 2222 (43.84%) were female patients, and 2846 (56.16%) were male patients. The most frequently identified were White (55.51%) patients, followed by Black/African American (22.49%) patients, multiracial/other (18.11%), and Asian (1.42%) patients (2.47% were missing). A total of 1091 (21.53%) Hispanic/Latino/a patients were identified, and 3788 (74.74%) patients did not (3.73% were missing). Patients most commonly had Medicare (43.23%), followed by private insurance (29.76%), Medicaid (23.15%), no insurance (2.15%), and other insurance (1.72%). A total of 318 (6.27%) patients lived in Maywood (zip code: 60153), and 4749 (93.71%) patients lived outside of Maywood (0.02% were missing). For the LUMC M2B inpatient bedside medication delivery program, 3008 (59.35%) patients were enrolled, and 904 (17.84%) patients were not (22.81% were missing). During inpatient admission, 2258 (44.55%) patients had a confirmed bedside medication delivery (25.85% were missing). A total of 447 patients, constituting 8.82% of the entire cohort, were readmitted within 30 days (Table 1).

**Table 1:** Patient demographics, bedside medication delivery, and inpatient outcomes Missing observations, n (%): Race: 125 (2.47), ethnicity: 189 (3.73), zip code: 1 (0.02), meds-to-beds enrollment: 1156 (22.81), last inpatient medication delivery status: 1310 (25.85), and confirmed bedside medication delivery: 1310 (25.85)

Variable	n (%)
Sex	
Female	2222 (43.84)
Male	2846 (56.16)
Race	
Multiracial/other	918 (18.11)
Asian	72 (1.42)
Black/African American	1140 (22.49)
White	2813 (55.51)
Ethnicity	
Hispanic/Latino/a	1091 (21.53)
Non-Hispanic/Latino/a	3788 (74.74)
Insurance	
Uninsured	109 (2.15)
Other	87 (1.72)
Medicaid	1173 (23.15)
Medicare	2191 (43.23)
Private	1508 (29.76)
Zip Code	
Maywood (60153)	318 (6.27)
Not Maywood	4749 (93.71)
Meds-to-Beds Enrollment	
Yes	3008 (59.35)
No	904 (17.84)
Last Inpatient Medication Delivery Status	
Canceled	410 (8.09)
Canceled – SNF/LTC	45 (0.89)
Delivered to the patient	2258 (44.55)
Fill completed	62 (1.22)
Fill in progress	46 (0.91)
No bedside medication delivery	904 (17.84)
Waiting on payment	33 (0.65)
Confirmed bedside medication delivery	
Yes	2258 (44.55)
No	1500 (29.60)
Hospital readmission	
Yes	447 (8.82)
No	4621 (91.18)

There was no significant association observed between M2B enrollment and 30-day hospital readmission (p=0.58). Readmission rates were comparable between patients who were (9.34%) and were not (8.74%) enrolled in the program. Insurance did demonstrate a significant association with hospital readmission (p<0.01). Around 10.95% of patients on Medicare were readmitted, whereas only 3.67% of uninsured patients were readmitted. Zip code was significantly associated with hospital readmission (p<0.01). Hospital readmission rates were significantly higher among patients living in Maywood (13.52%) than among those living elsewhere (8.51%). Hospital readmission was not significantly associated with sex (p=0.23), race (p=0.29), ethnicity (p=0.51), or confirmed bedside medication delivery (p=0.16) (Table 2). 

**Table 2:** Hospital readmission by patient demographics and meds-to-beds participation *Significant at α=0.05 level †Pearson’s Chi-square test p-values

Variable (%)	30-day hospital readmission		Χ^2^	p†
	Yes	No		
Sex			1.43	0.23
Female	184 (8.28)	2038 (91.72)		
Male	263 (9.24)	2583 (90.76)		
Race			3.74	0.29
Multiracial/other	92 (10.02)	826 (89.98)		
Asian	5 (6.94)	67 (93.06)		
Black/African American	111 (9.74)	1029 (90.26)		
White	235 (8.35)	2578 (91.65)		
Ethnicity			0.43	0.51
Hispanic/Latino/a	105 (9.62)	986 (90.38)		
Non-Hispanic/Latino/a	340 (8.98)	3448 (91.02)		
Insurance			27.22	<0.01*
Uninsured	4 (3.67)	105 (96.33)		
Other	6 (6.90)	81 (93.10)		
Medicaid	100 (8.53)	1073 (91.47)		
Medicare	240 (10.95)	1951 (89.05)		
Private	97 (6.43)	1411 (93.57)		
Zip Code			9.32	<0.01*
Maywood (60153)	43 (13.52)	275 (86.48)		
Not Maywood	404 (8.51)	4345 (91.49)		
Meds-to-Beds Enrollment			0.30	0.58
Yes	281 (9.34)	2727 (90.66)		
No	79 (8.74)	825 (91.26)		
Confirmed Bedside Medication Delivery			1.98	0.16
Yes	191 (8.46)	2067 (91.54)		
No	147 (9.80)	1353 (90.20)		

M2B enrollment was significantly associated with sex (p=0.02), race (p=0.03), ethnicity (p<0.01), and patient insurance (p<0.01). M2B enrollment rates were significantly higher for male patients than for female patients (78.24% vs. 75.10%), and significantly higher for Hispanic/Latino/a patients than for Non-Hispanic/Latino/a patients (80.94% vs. 75.82%). Asian patients generally demonstrated higher M2B enrollment rates than non-Asian patients. Uninsured patients and patients on Medicaid generally demonstrated higher M2B enrollment rates than patients with alternative insurance types. M2B enrollment was not significantly associated with zip code (p=0.18) (Table 3). Demographic patient characteristics stratified by zip code are described in Table 4. Length of stay was significantly associated with both 30-day hospital readmission (p<0.01) and M2B enrollment (p<0.01) (Table 5). 

**Table 3:** Meds-to-beds enrollment by patient demographics *Significant at α=0.05 level †Pearson’s Chi-square test p-values

Variable (%)	Meds-to-beds enrollment		Χ^2^	p†
	Yes	No		
Sex			5.29	0.02*
Female	1261 (75.10)	418 (24.90)		
Male	1747 (78.24)	486 (21.76)		
Race			9.03	0.03*
Multiracial/other	576 (79.78)	146 (20.22)		
Asian	45 (84.91)	8 (15.09)		
Black/African American	670 (77.91)	190 (22.09)		
White	1637 (75.26)	538 (24.74)		
Ethnicity			9.73	<0.01*
Hispanic/Latino/a	688 (80.94)	162 (19.06)		
Non-Hispanic/Latino/a	2211 (75.82)	705 (24.18)		
Insurance			97.01	<0.01*
Uninsured	77 (84.62)	14 (15.38)		
Other	57 (76.00)	18 (24.00)		
Medicaid	813 (88.37)	107 (11.63)		
Medicare	1234 (73.50)	445 (26.50)		
Private	827 (72.10)	320 (27.90)		
Zip Code			1.81	0.18
Maywood (60153)	170 (73.28)	62 (26.72)		
Not Maywood	2837 (77.11)	842 (22.89)		

**Table 4:** Patient demographics and insurance status by zip code

Variable	Zip code	
	Maywood (60153) (%)	Not Maywood (%)
Sex		
Female	165 (51.89)	2057 (43.31)
Male	153 (48.11)	2692 (56.69)
Race		
Multiracial/other	30 (9.43)	888 (18.70)
Asian	0 (0.00)	72 (1.52)
Black/African American	247 (77.67)	893 (18.80)
White	34 (10.69)	2778 (58.50)
Ethnicity		
Hispanic/Latino/a	47 (14.78)	1043 (21.96)
Non-Hispanic/Latino/a	264 (83.02)	3524 (74.21)
Insurance		
Uninsured	11 (3.46)	97 (2.04)
Other	1 (0.31)	86 (1.81)
Medicaid	93 (29.25)	1080 (22.74)
Medicare	158 (49.69)	2033 (42.81)
Private	55 (17.30)	1453 (30.60)

**Table 5:** Length of stay by 30-day hospital readmission and meds-to-beds enrollment *Significant at α = 0.05 level †Wilcoxon rank sum test p-values IQR, interquartile range.

Variable	Yes		No		Z	p†
	n	Length of stay (days) - median (IQR)	n	Length of stay (days) - median (IQR)		
30-day hospital readmission	447	5.67 (3.84-8.92)	4616	4.43 (2.80-7.30)	6.84	<0.01*
Meds-to-beds enrollment	3004	5.28 (3.29-8.25)	904	4.22 (2.81-6.64)	-8.33	<0.01*

## Discussion

Transitions of care place patients at elevated risk for adverse events that may result in hospital readmission and increased risk of morbidity or mortality [10]. Medication non-adherence is one such event. Barriers to medication adherence at the time of discharge can be extensive; well-documented patient-reported examples include not having medications dispensed before discharge, increased costs of new medications, poor communication between patient and care teams regarding medication changes and reconciliation with prior medications, difficulty navigating to a community pharmacy after discharge, and discharge to a care facility that is unable to dispense appropriate medications [11,12]. We hypothesized that a bedside medication delivery program should ensure that more patients leave with medications in hand, have the chance to speak with pharmacy representatives about their medications before discharge, and eliminate the need to travel to a community pharmacy immediately following discharge. We assessed the impact of our program primarily through 30-day hospital readmissions. 

In our study, enrollment in the M2B program was not significantly associated with a reduced risk of 30-day hospital readmission (p=0.58). The association between 30-day hospital readmission and confirmed bedside medication delivery approached but did not reach significance (p=0.16). Analysis of programs with similar structures remains inconclusive. There is some evidence that select patient populations may benefit from bedside medication delivery: Stedge et al. found that 30-day readmissions were significantly reduced in patients with no comorbidity burden or very high comorbidity burden, when stratified by the Charlson Comorbidity Index score [13]. Another large study by Islam et al. (n=45,546) found that 30-day hospital readmission rates were significantly lower among some surgical patients who received bedside medication delivery but not in the overall cohort [14]. Only two studies, by Lash et al. and Kirkham et al., found that bedside medication delivery decreased 30-day readmission rates in the entire patient population. One of these studies noted a small cohort size (n=344), the other featured a large cohort (n=19,659) and notably included a follow-up telephone call to patients after discharge [15,16]. Four other studies, ranging from single-center to statewide database analyses, did not find a significant reduction in 30-day hospital readmissions after implementation of a bedside medication delivery program [17-20]. These findings and the lack of sufficient data surrounding the generalizability of such programs were reinforced in the only published meta-analysis on this topic [9].

We further reviewed the available literature for strategies that may improve our own and similar programs. A 2013 survey of 478 hospitals by Bradley et al. analyzed 93 individual practice strategies for reducing hospital readmissions, which did not include bedside medication delivery. The only individual strategy that reduced readmissions in isolation was discharging patients with follow-up appointments already in place. It was also found that hospitals that bundled three or more strategies into a comprehensive intervention saw reduced readmissions [21]. This reinforces the work of Kirkham et al., who found that bedside medication delivery reduced readmissions when paired with post-discharge telephone follow-up [16]. We hypothesize that while bedside medication delivery, as presently implemented, may not be a broadly effective strategy to reduce readmissions in isolation, future studies should examine ways to bundle it into more comprehensive multistep interventions. 

Demographic characteristics, including patient sex, race, and ethnicity, helped us pinpoint particular patient groups with higher or lower enrollment rates in the program; none were associated with hospital readmission. Only patient insurance status was associated with both program enrollment and readmission. Patients with Medicare had higher readmission rates and lower M2B enrollment rates. Patients who were uninsured had among the lowest readmission rates but also had among the highest M2B enrollment rates. The distribution of insurance providers in our cohort was similar to that reported in the reviewed studies that made this data available. Among these studies, Witcraft et al. found patients with Medicare or Medicaid to be at higher risk of readmission; Segel et al. found the risk to be highest among uninsured patients [17,20]. Large trials examining national-level databases on hospital readmissions have found rates to vary with insurance status and to be highest among patients with Medicare and the lowest among the uninsured [22]. It has been theorized that the lack of health insurance itself is a barrier to seeking care that may lead to a readmission, and that patients on Medicare are more likely to experience complex, multifactorial comorbidities or end-of-life care. How bedside medication delivery after program enrollment may impact patients with a specific insurance status remains unclear and is likely confounded by socioeconomic and comorbid factors such as these. 

Hospital readmission was also significantly associated with zip code. We specifically analyzed patients with a confirmed address in Maywood, IL, zip code 60153, as our center is located in and closely tied to this community, which is recognized as medically underserved by the Health Resources and Services Administration. To our knowledge, no related study has specifically analyzed a patient population that is known to be underserved. These patients had higher readmission rates despite having similar rates of enrollment in the program. In Maywood, Black and non-Hispanic patients were most commonly identified. Among these patients, 46% were uninsured compared with 2.04% of the non-Maywood population. The majority had Medicare or Medicaid. These patients likely face many of the barriers to medication adherence after discharge noted above and may specifically benefit from focused multistep interventions moving forward. 

Our study has limitations that should be noted. First, this was a cross-sectional single-center observational study. We did attempt to consider our findings within the context of those noted previously for similar interventions. It is possible that selection bias affected the patients enrolled in M2B, as their decision to enroll may have been influenced by factors outside of the studied variables. As our review of medical records was limited to our center, we were not able to account for readmissions to outside hospitals. We were unable to stratify patients by the medical or surgical service they were admitted to or by their indication for hospital admission because the M2B service operates by hospital unit and is independent of the primary care team or the indication for hospitalization. Our primary endpoint was 30-day hospital readmission, but we were otherwise unable to specifically assess whether medication adherence after discharge was different for patients enrolled in the program. We also note that substantial residual confounding from patient comorbidity burden may be present, given the association between Medicare status and length of stay. Finally, it is difficult to assess the generalizability of our findings. This includes hospital-specific factors, as some hospitals may not have an on-site outpatient pharmacy, adequate pharmacy staffing to support such a program, or institutional workflow differences among the various staff members involved in these programs. 

## Conclusions

We sought to determine whether bedside medication delivery by pharmacy staff before hospital discharge was associated with reduced 30-day hospital readmissions via the implementation of the M2B program, using data derived from quality improvement practices at our center. We did not find that our program, as presently implemented, was associated with reduced hospital readmissions, but we were able to identify certain patient groups for which the program may not be sufficiently serving. Prior studies have concluded that more data on bedside medication delivery programs is needed. After analyzing those previously reported, we believe that future interventions may most significantly benefit from bundling multiple strategies in the peri-discharge period. The impact of patients' insurance status on enrollment in bedside medication delivery programs at other centers, and its relationship to hospital readmission, also warrants further investigation.
